# Topological dual and extended relations between networks of clathrate hydrates and Frank-Kasper phases

**DOI:** 10.1038/s41467-023-36242-4

**Published:** 2023-02-03

**Authors:** Yong Chen, Satoshi Takeya, Amadeu K. Sum

**Affiliations:** 1 Phases to Flow Laboratory, Chemical & Biological Engineering Department, Colorado School of Mines, 1500 Illinois Street, Golden, CO 80401 USA; 2 Guangzhou Institute of Energy Conversion, Chinese Academy of Sciences, No.2, Nengyuan Road, Wushan, Tianhe District, Guangzhou, 510640 Guangdong PR China; 3 CAS Key Laboratory of Gas Hydrate, Guangzhou, 510640 Guangdong PR China; 4 Guangdong Provincial Key Laboratory of New and Renewable Energy Research and Development, Guangzhou, 510640 Guangdong PR China; 5 State Key Laboratory of Natural Gas Hydrate, Beijing, 100028 PR China; 6 National Metrology Institute of Japan (NMIJ), National Institute of Advanced Industrial Science and Technology (AIST), Central 5, Higashi 1-1-1, Tsukuba, 305-8565 Ibaraki Japan

**Keywords:** Physical chemistry, Structure of solids and liquids, Materials chemistry

## Abstract

Clathrate hydrates are a class of ordered structures that are stabilized via the delicate balance of hydrophobic interactions between water and guest molecules, of which the space-filling network of hydrogen-bonded (H-bonded) water molecules are closely related to tetrahedrally close-packed structures, known as Frank-Kasper (FK) phases. Here we report an alternative way to understand the intricate structures of clathrate hydrates, which unveils the diverse crystalline H-bonded networks that can be generated via assembly of one common building block. In addition to the intrinsic relations and pathways linking these crystals, we further illustrate the rich structural possibilities of clathrate hydrates. Given that the topological dual relations between networks of clathrate hydrates and tetrahedral close-packed structures, the descriptors presented for clathrate hydrates can be directly extended to other ordered materials for a more thorough understanding of their nucleation, phases transition, and co-existence mechanisms.

## Abstract (web summary)

Clathrate hydrates are topological duals of Frank-Kasper phases. Here, the authors employ MD simulations to provide an alternative way to explore the intrinsic structural relationships of H-bonded networks of clathrate hydrates and other unrelated ordered materials.

## Introduction

The science of water and ice still brings surprises to this day. As such, it is expected that the science of clathrate hydrates, which are solid solutions of water and gas, reveals even more intricacies from the delicate balance of hydrophobic interactions for the space-filling networks of hydrogen-bonded water molecules^1^. Among them, three structures are the cornerstones of clathrate hydrates, denoted as Type I, II, and H^2–4^. However, the known crystalline structures of clathrate hydrate are not unique. A majority of clathrate hydrates structures are closely related to a class of intermetallic structures, known as FK phases.

**Fig. 1:**
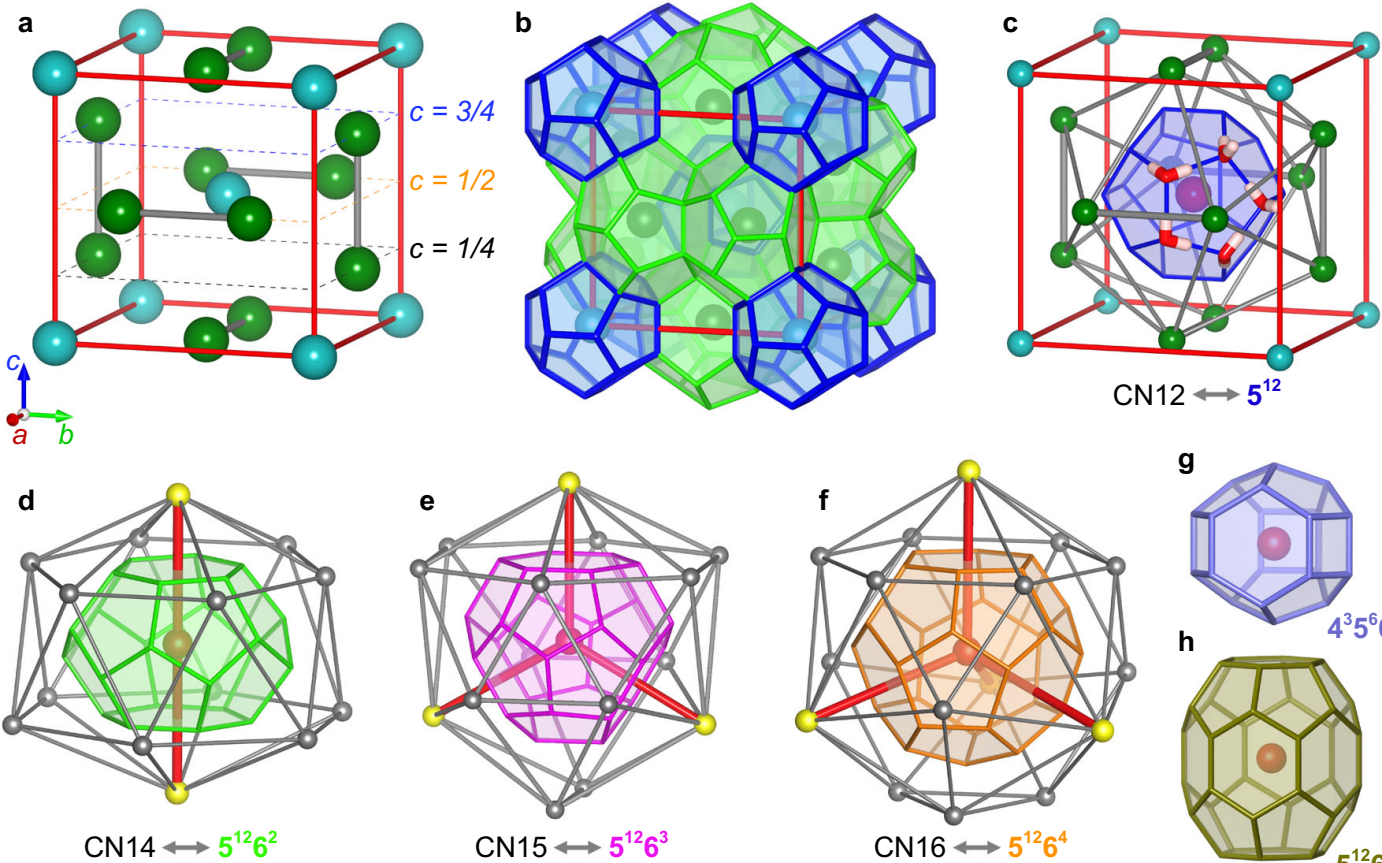
FK phases and clathrate hydrates structures. *A*15 phase **(a)** and Type I clathrate hydrates **(b)** as examples illustrating the TCP and H-bonded network of water molecules. The cyan and dark green spheres represent central nodes of CN12 and CN14, respectively. Dash lines in **(a)** indicate nodes at different *c* direction layer, 1/4 (black), 1/2 (orange), and 3/4 (blue). **c** The CN12 polyhedron (gray lines) and 5^12^ cage (blue, means 12 pentagonal faces) contained in the *A*15/Type I lattice. Red and white rods are water molecules specifically displaying a H-bonded pentagonal face. **d**–**f** Topological dual relationship between H-bond cages and CN polyhedrons (gray lines). There are two kinds of nodes in CN polyhedrons, five-fold (gray) and six-fold (yellow). The red lines connect central node (red) and six-fold nodes for a better illustration. The 5^12^6^2^, 5^12^6^3^, and 5^12^6^4^ are displayed in green, magenta, and orange, respectively. **g**, **h** H-bond cages in Type H clathrate hydrates, 4^3^5^6^6^3^ (purple) and 5^12^6^8^ (olive). The cages in the figures to follow will have the same color scheme used here.

FK phases are tetrahedrally close-packed (TCP) structures, which are observed in a broad range of physical scales, from a few angstroms in alloys^5,6^ to mesoscopic-scale in various self-assembly of soft matter^7–15^. Generally, the TCP networks are described by the combination of four polyhedrons with only triangular faces^16^ (see Fig. 1). The icosahedron with coordination number 12 (CN12) interweaves with at least one of CN14, CN15, or CN16 forming diverse crystalline networks (27 types known^17^) that contains exclusively tetrahedral interstices. Instead of directly considering the densely packed framework as for the FK phases, the clathrate hydrate community focuses on its topological dual (Wigner–Seitz or Voronoi cells), that is, the network formed of H-bonded water molecules. Considering clathrate hydrates from the close packing perspective, the guest molecules at the center of the cages, or just the center position for empty cages, correspond to the TCP nodes in the FK phases, while the water molecules fill the tetrahedral interstices and are connected through H-bond forming a complex network (Fig. 1a–c). The network of H-bonded water molecules partition the space into a series of cavities (denoted H-bond cages) where guest molecules encaged, in principle, are the topological dual of CN polyhedrons (Fig. 1c–d). However, it is not uncommon for cages in clathrate hydrates to be empty which renders the non-stoichiometric characteristic of clathrate hydrates^1^, that is, the non-integer number of guest molecules to the number of water molecules in the clathrate structure. This unusual fact means that even though the guest molecules are insufficient to define the TCP, the H-bonded water network still remains stable and has FK ordered structure in the form of topological duals. The collective structural stabilization not entirely relied on TCP differs clathrate hydrates from other conventional FK phases, which is one of the unique and beneficial aspect for structural diverse based on clathrate hydrate structures. Nevertheless, from the perspective of topological duality, clathrate hydrate is a valuable platform for structurally probing relations among FK phases, since their diverse crystals appear on the same physical scale, which was previously only considered mathematically (Yarmolyuk-Kripyakevich’s empirical formula for describing FK phases^18–20^). Moreover, H-bond cages have an advantage over the CN polyhedrons when understanding detailed 3D structures, as the connectivity between the former is simpler and more defined than the latter – see a detailed discussion in Supplementary Note 1. We also note that the topological duals of the Type H clathrate hydrate follow the CaCU_5_ prototype^21^ in the intermetallic structures (some studies classify it as quasi-FK phase^22^), which indicates clathrate hydrates may provide more insight into the densely-packed structures, not limited to TCP.

In this work, based on a thorough investigation of the H-bond network of clathrate hydrates, including the three dominant and two relative rare crystals (Type HS-I^23,24^ and TS-I^25^, their counterparts in FK are *Z* and *Sigma* phases), we propose an alternative way to understand these intricate 3D networks. In addition to unveiling the intrinsic relations and pathways linking these crystals, we further illustrate the richness in structural possibilities of clathrate hydrates. Importantly, given the topological dual relations discussed above, our findings of H-bonded networks of clathrate hydrates can be directly extended to other apparently unrelated materials and kinetic processes in different physical scales.

## Results

### Basic building blocks

**Fig. 2:**
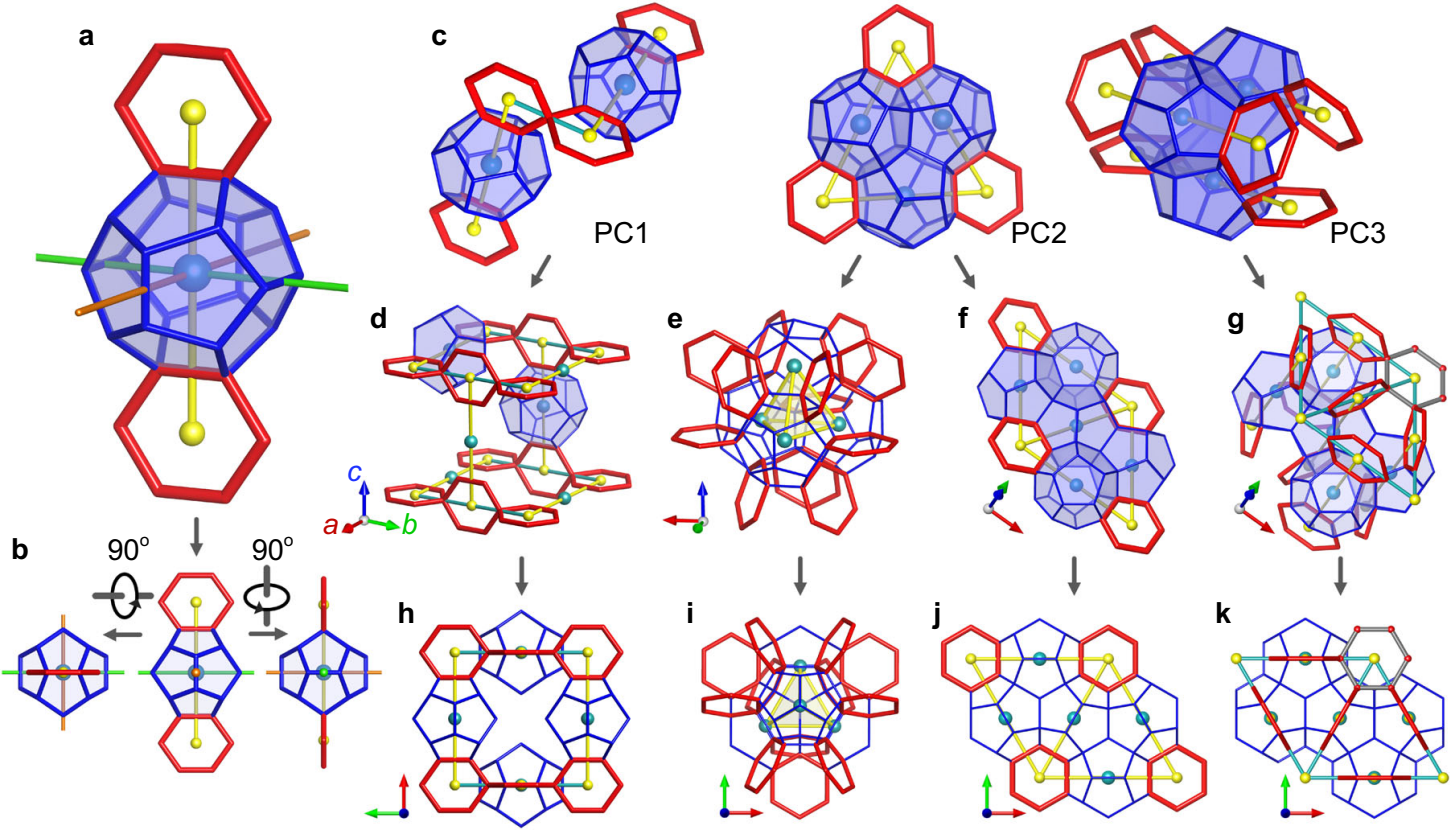
Building blocks. **a** The basic building block: 5^12^ cage and hexagonal rings are displayed in blue and red lines, respectively, and their respective centers are cyan and yellow spheres. The green and orange lines are auxiliary lines for illustrating the 3D structure. The images in **(b)** show its projection view along other directions. The basic building block is slightly different in different structures, see Supplementary Note 2. **c** Three primitive connectivities of the basic building blocks, PC1–3. **d** 3D structure of CBD, some 5^12^ cages are hidden for clarity. **e** Pyramid building block. **f** R-A building block. **g** R-B building block. The gray lines manifest the six-fold symmetry. **h**–**k** Projection view along *c* direction for corresponding **(d**–**g)** structures.

Conventionally, the crystalline clathrate hydrates structures are described by the combination of elementary cages as listed in Supplementary Table S1, mindful that numerical descriptions cannot express the steric connectivities. Through careful deconstruction of the H-bond network of clathrate hydrates, we replaced the well-established elementary cages description to define one common building block that is identified in all crystal structures discussed in this work: 5^12^ cage with two hexagonal rings. As shown in Fig. 2a, the two hexagonal rings lie in the same plane and each share an edge with the 5^12^ cage. The line connecting the center of the hexagonal rings passes through the center of the 5^12^ cage, and it will be called centerline. We summarize three primitive connectivities of this basic building block (BBB) in crystalline clathrate hydrates, as shown in Fig 2c: (PC1) two BBBs are connected via a node in the hexagonal rings (a water molecule) and their centerline are perpendicular; (PC2) BBBs are connected by sharing hexagonal rings, meanwhile their 5^12^ cages merge through pentagonal faces, which leads to an equilateral triangle building block; (PC3) the arrangement of the 5^12^ cage is the same as in PC2, while their centerlines are parallel. Following these three primitive connectivities, other more complex building blocks can be derived to assemble diverse crystalline H-bonded networks. The network extends exclusively following the PC1 forming a cube building block (CBD, Fig. 2d). The 2D tiling of the equilateral triangle building block has six-fold symmetry, which is represented by a rhombic block (R-A, Fig. 2f); similarly, PC3 derives another type of rhombic block (R-B, Fig. 2g). Moreover, the PC2 interlaced in 3D forms a pyramid building block (Fig. 2e)—a detail structure is shown in Supplementary Fig. 4.

All the other elementary cages can be generated via symmetry operations of these building blocks (Supplementary Fig. 5), which means the crystalline network of H-bonded water molecules can be generated from this proposed basic building block. In other words, the diverse crystal structures of clathrate hydrates are geometrically different stacking patterns of one common building block. This interesting fact and the building blocks lead us to discover pathways among different crystals and further unveil other structural complexities. It is noteworthy that “pathways” in this work mean different structures that connect seamlessly in the solid state, not the transformation among structures through local rearrangement of atoms.

### Pathways and structure complexity among Type I, HS-I, and TS-I

**Fig. 3:**
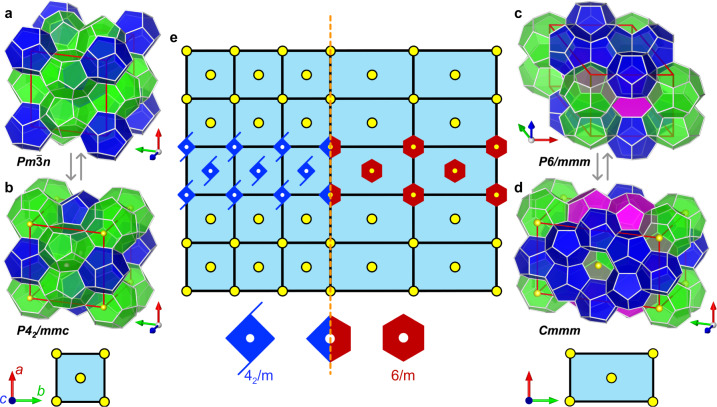
The pathway between Types I and HS-I. **a, b** The 3D structures of CUC and AUC of Type I, respectively. **c, d** The 3D structures of CUC and AUC of Type HS-I, respectively. The images below **(b, d)** are 2D tiles representation of AUCs of Type I and HS-I, respectively. The yellow nodes indicate the rotation symmetry axes in the unit cells. **e** Schematic diagram shows Type I and HS-I merge through their AUCs. ‘6/m’ means six-fold rotation axis perpendicular to a mirror plane, and similarly for ‘4_2_/m’. The orange dash line indicates the crystal transition region.

Type I clathrate hydrates are the most abundant in the Earth’s natural environments^1,24,26^, and its FK counterpart, the *A*15 phase, also widely occurs in alloys and soft matter^9,27^. A unit cell of Type I is composed of two 5^12^ cages and six 5^12^6^2^ cages with cubic $Pm\overline{3}n$ space group. From the viewpoint of the building blocks, the Type I network can be depicted by the CBD (Fig. 2d). Focusing on the hexagonal rings present on the corner of the CBD, it is clear that their centers are equivalent points that can be generated via three independent translational movements (see Supplementary Fig. 6), thus the CBD satisfies the definition of 3D lattice (unit cell) in space group. As the duplication of CBD unit cells can generate the Type I structure, one may take for granted that this primitive cubic lattice falls into the $Pm\overline{3}n$ space group, but it is not the case. The CBD cell is an alternative unit cell (AUC) of the Type I structure, and can be obtained by shifting the periodic box of the conventional unit cell (CUC) by half unit cell, that is, scan 0.5–1.5 of the CUC (see Fig. 3a, b and Supplementary Fig. 7). Consequently the three-fold symmetry axes (more exactly, the $\overline{3}$ axis) along the body diagonals of the CUC, is not along the body diagonals of the AUC, as also shown by the arrangement of the 5^12^ cages (Supplementary Fig. 6e). This renders the symmetry of nodes in the AUC different from that of the CUC, even though the unit cell remains cubic and the unit cell content is unchanged (Supplementary Fig. 7 and Supplementary Table 3). We identify the symmetry of the CBD as tetragonal *P*4_2_/*mmc*, a lower symmetry version of the $Pm\overline{3}n$. Normally, there is no reason to describe Type I (and *A*15) structure as *P*4_2_/*mmc*. However, the 4_2_ screw symmetry explicitly expressed by the CBD cell, together with its cell geometry (arrangement of nodes), opens up pathways for linking different crystals, an insight central but not appreciated prior to this work.

Type HS-I (*Z* phase) structure is a hexagonal crystal with *P*6/*mmm* space group, which unit cell can be established by the stacking of 2D hexagonal lattice (represented by R-A building block). Beside the conventional rhombic cell, the 2D hexagonal lattice can be treated as centered rectangular cell^28^. This equivalent conversion leads to the orthorhombic AUC of Type HS-I with *Cmmm* space group (Supplementary Fig. 8 and Supplementary Table 4). The *Cmmm* returns to the orthogonal coordinate system and it has the same surface with *P*4_2_/*mmc* (the face orthogonal to the *ac* plane, see Fig. 3b, d). Figure 3 illustrates two distinct lattices merged through their AUCs, where the six-fold rotation axes implicit in *Cmmm* lattice coincides with the 4_2_ screw axes of the *P*4_2_/*mmc* lattice. On one side it retains the 4_2_ screw symmetry, while in the opposite side, the axes switch to the six-fold rotation symmetry, and as such, the structural change smoothly occurs without changing the phase. Moreover, considering the FK phases counterparts, *A*15 and *Z* phases, this result indicates that despite the structural change that occurs, the TCP identity is still continuous, even at the joints. The coincidence of six-fold and 4_2_ screw axis does not create new symmetry, so the pathway between Types I and HS-I structures cannot be assigned a space group, while it is structurally compatible. In principle, the differences between Types I and HS-I are relatively minor, as they both can be generated through one common building block with just a different type of rotation axis: the six-fold rotation axis builds Type HS-I network while the 4_2_ screw axis sets up the Type I network (Supplementary Fig. 9). Both rotation operations result in a column of 5^12^6^2^ and create a common cross-section, which is the fundamental reason for the structural compatibility between Types I and HS-I.

**Fig. 4:**
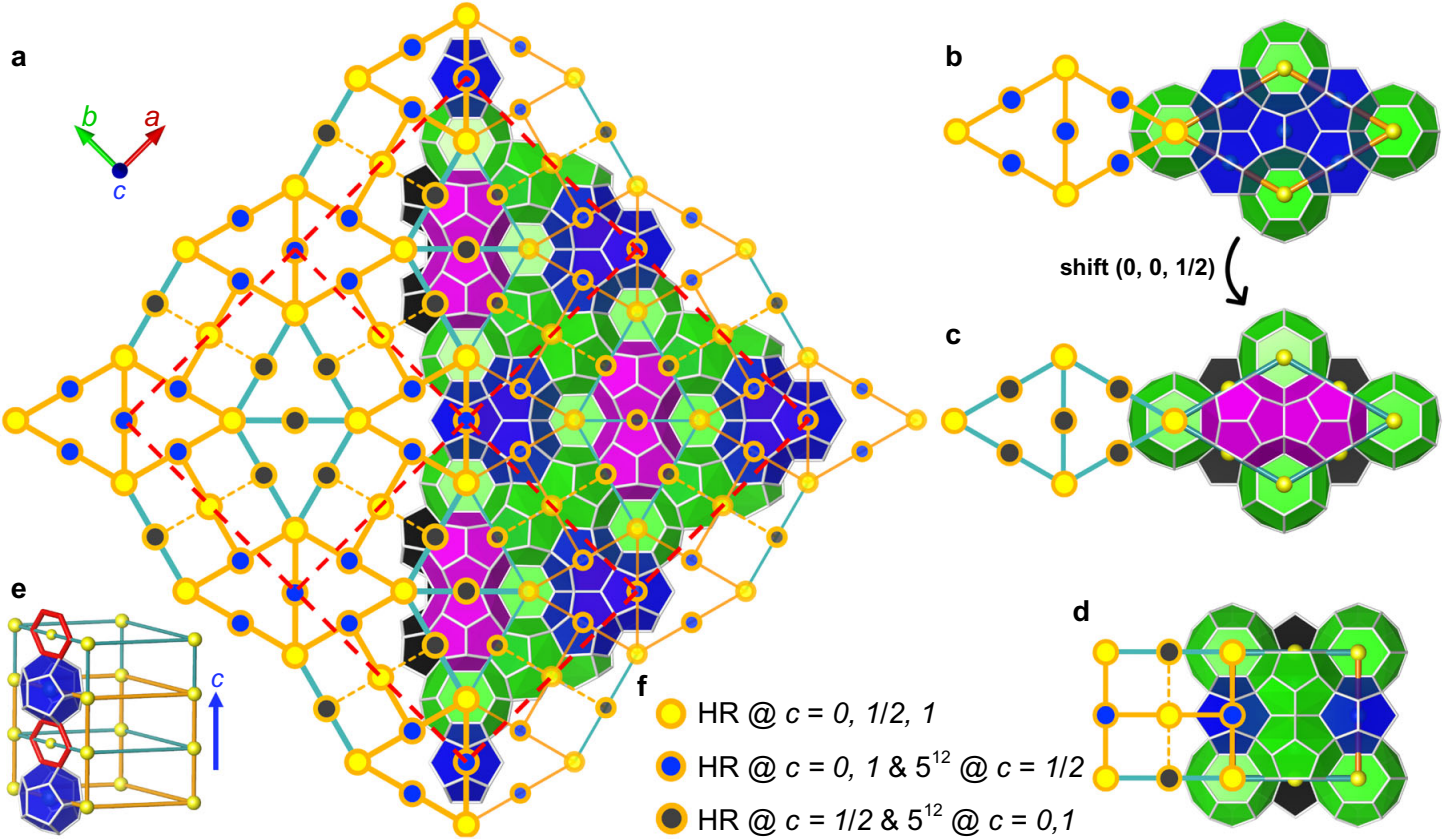
Tessellation of Type TS-I by Types I and HS-I. **a** 2 × 2 unit cells of Type TS-I viewed along the [001], the red dash line shows the unit cell boundary. The view cuts it open along the [110]; the left part shows the structure in 2D tiles and the right part details its cages. To distinguish 5^12^ cages in different layers, the 5^12^ cages at *c* = $\frac{1}{2}$ are black, and those at *c* = 0, 1 are blue. **b**–**d**, Two types of *P*6/*mmm* unit cells of Type HS-I **(b, c)** and *P*4_2_/*mmc* unit cell of Type I **(d)** with their corresponding 2D version. The 3D structures in **(a**–**d)** are simplified as dots, see annotations in **(f)**, which indicates the position of hexagonal rings (HR) and 5^12^ cages. **e**, Relationship between cells shown in **(b, c)**. The yellow spheres are centers of hexagonal rings. Blue arrow indicates *c* direction.

Type TS-I (*P*4_2_/*mnm*, *Sigma* phase) can be interpreted as another manifestation of the compatibility between Types I and HS-I. As shown in Fig. 4, Type TS-I structure can be tiled with Types I and HS-I. Two different appearances of rhombic prism cells are essentially equivalent, and they can be mutually converted by translating half unit cell along the *c* direction. The collective shift happens along the rotation axis, so nothing changes in terms of symmetry, just a matter of the choice of the basal plane. The 4_2_ symmetry of *P*4_2_/*mmc* clearly illustrates the complex connectivity among these three building blocks, that is, two adjacent surfaces (intersected along the 4_2_ axis) differ by $\frac{1}{2}$ unit cell along the 4_2_ axis direction. However, following this connectivity, the obtained structure does not just point to Type TS-I, as some combinations may lead to other unexpected ordered structures – quasicrystals, which will be discussed later.

**Fig. 5:**
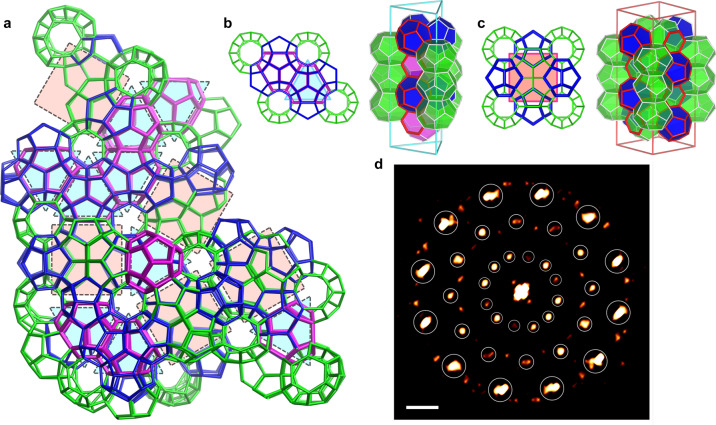
Structural complexity in the mixture of Types I, HS-I, and TS-I. **a** A cluster captured from MD simulations. Local order or so-called crystallite of Types I, HS-I, and TS-I can be recognized in it. It also can be understood as a random tiling of squares and triangles, which is closely reminiscent of dodecagonal quasicrystals. Supplementary Movie 1 shows the nucleation process of this cluster. **b, c** The equilateral triangle and the square tile corresponds to half *P*6/*mmm* unit cell of Type HS-I and *P*4_2_/*mmc* unit cell of Type I, respectively; the image on the right show their 3D structure. The red lines highlight the common structure/surface in triangular and square prisms to illustrate their connectivity. **d** The FFT of **(a)** shows 12-fold symmetry highlighted by circles. Scale bar: 0.5 Å^−1^.

Given that Type TS-I can be understood as a specific mixture of Type I and HS-I, thus, predictably, these three crystals can together build one phase. Including but not limited to the so-called polycrystals, that each crystal has its domain and concatenate together. Figure 5 shows an example captured from our molecular dynamics (MD) simulations to further present the structural complexity that the mixture of Type I, HS-I, and TS-I could manifest. Through different partitions of the local region, traces of the three aforementioned crystals can be recognized in Fig. 5a, and Supplementary Movie 1 shows its nucleation process. Obviously there is no translational symmetry in this cluster, nevertheless, it is improper to classify it as amorphous solid without further analysis. Leaving aside complete unit cells, the Fig. 5a cluster can be considered as the random tessellation of squares and equilateral triangles, which are the 2D substitutions for *P*4_2_/*mmc* and half cell of *P*6/*mmm* (Fig. 5b, c), respectively. Analogous patterns are found in dodecagonal quasicrystals of alloys^29^ and soft matter^7,10^. We further conducted the fast Fourier transform (FFT) along the high-symmetry direction of the captured cluster, which clearly shows 12-fold symmetry and confirms the quasiperiodicity (Fig. 5d). Thus, there is a strong possibility that H-bonded water molecules formed under certain conditions and with certain guest molecules can manifest quasi-periodic order, which is an original concept for clathrate hydrates, the cluster captured in our simulations is the first identified evidence of this possibility. The *P*4_2_/*mmc* and half unit cell of *P*6/*mmm* as building blocks clearly shows 2D tilings stretching to 3D, and the connectivities among building blocks shown in Fig. 4 are helpful for describing the complex structure in relatively simple terms.

### Pathways and structure complexity among Types II, HS-I, and H

The network of Type II structure ($Fd\overline{3}m$, *C*15 phase) can be set up exclusively by the pyramid building blocks, connected by sharing one node (a 5^12^ cage), resulting in a cubic crystal symmetry, as shown in Supplementary Figs. 10a,b. The inverted connected pyramid building blocks automatically generates a series of layers of R-A building blocks with *p*6 symmetry. Interestingly, the inverted connected pyramids means that the six-fold symmetry axes of the upper *p*6 monolayer coincide with the three-fold symmetry axes of the lower *p*6 monolayer (see Supplementary Fig. 10). Only the three-fold symmetry survives in this case, and this special offset stacking of 2D hexagonal lattice indicates the cubic Type II can be converted to a trigonal lattice^28^. We conduct the coordinate system conversion based on the original cubic lattice to obtain the trigonal alternative unit cell with $R\overline{3}m$ space group. It has two versions, one prismatic cell and one rhombohedral cell: they are equivalent, just different expressions of the three-fold symmetry (Supplementary Fig. 10 and Supplementary Table 5).

The lattice produced by the duplication of the AUC is identical to the one produced by the CUC. Just as one always can find different ways to describe a lattice, it is the nature of symmetry, the difference is the degree of symmetry among these descriptions. It should be noted that the space group and unit cell are just tools for understanding infinite crystal lattices rather than a template or instructions for crystal growth. In reality, crystals are finite and they neither have to build complete unit cells nor have to show the highest symmetry during growth. Indeed, the $R\overline{3}m$ cannot compare with $Fd\overline{3}m$ in terms of symmetry, but it clarifies the layered characteristic of the Type II clathrate hydrate network. The immediate implication of this interpretation is that unexpected morphology may be observed during nucleation and crystal growth. In the process of clathrate hydrate growth, typically cubic crystals (Type II) are expected but it is not uncommon to observe hexagonal or trigonal patterns, as a recent experimental work probably captured this phenomenon^30^.

**Fig. 6:**
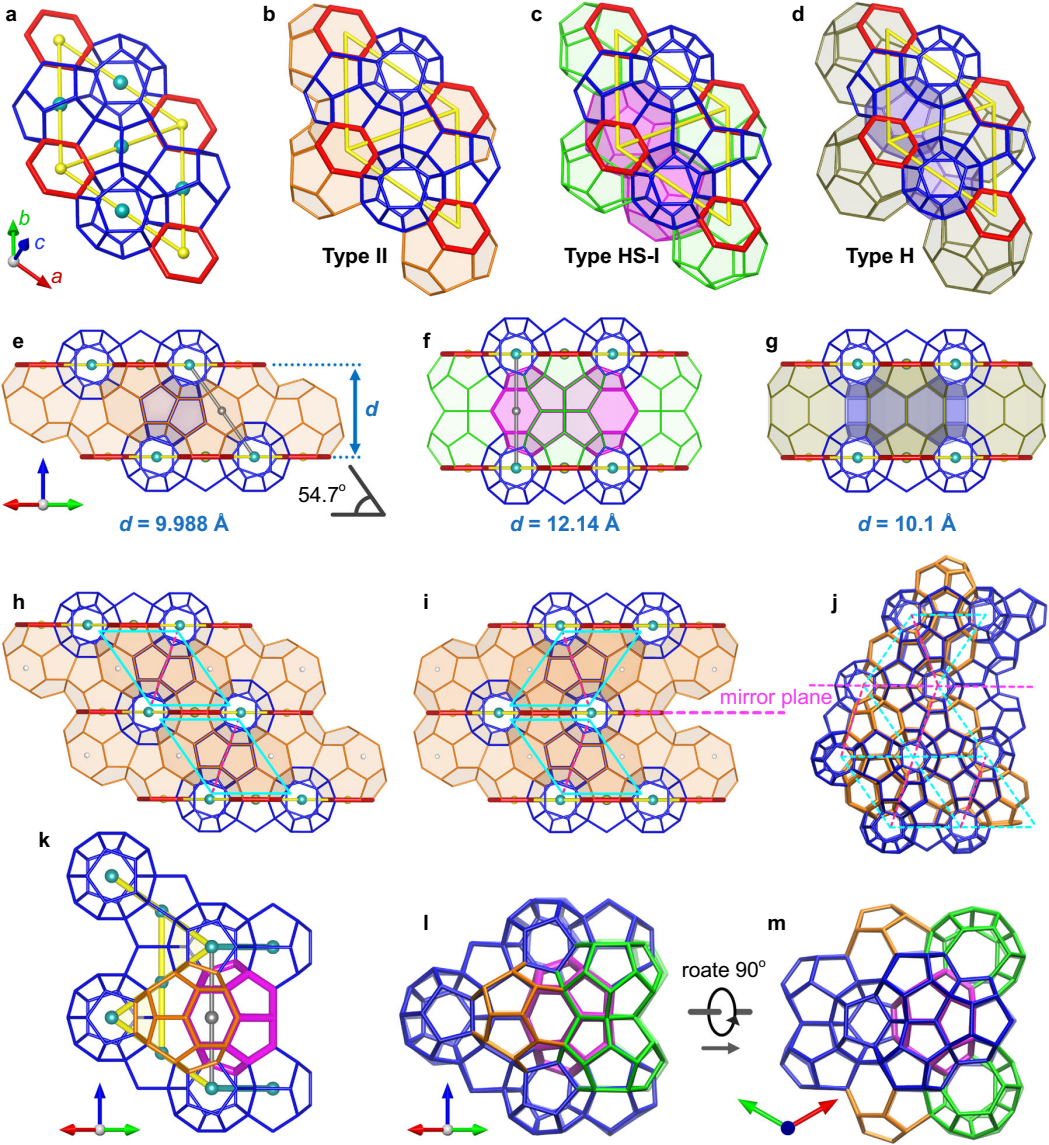
Intrinsic relation among Types II, HS-I, and H. **a**–**d** The same *p*6 monolayer **(a)** is present in three crystals (represented by R-A). The center of hexagonal rings and 5^12^ cages are hidden in **(b**–**d)** for clarity. **e**–**g** Interlayer structure of three crystals, viewed along the [110] with *d*-spacing annotated. The offset angle in the layered Type II **(e)** is 54.7° (Supplementary Fig. 10). The gray lines in **(e, f)** indicate the same structure in Types II and HS-I. **h, i** Changing the offset orientation results in a distinct structure. The offset orientation is consistent in **(h)**, and it is opposite in **(i)**, as indicated by the cyan parallelograms and the arrangement of hexagonal faces (small white spheres). **j** An example captured from MD simulation as evidence. **k** Schematic illustration of the Types II and HS-I merge/transit through the common interlayer structure. **l, m** An example captured from MD simulation as evidence.

For a better illustration of structure complexity, we choose the two offset *p*6 monolayers versions of the unit cell to represent the layered Type II. Types HS-I and H can be constructed by the stacking of the *p*6 monolayer represented by R-A building blocks as well, so the difference among these three (II, HS-I, H) crystals is the interlayer structures (Fig. 6a–g). Through the same monolayer, these three crystals can alternatively grow in any order. Of course, certain stacking combinations can be crystalline as long as the three-fold or six-fold symmetry is retained. A more intractable scenario is that the layers are stacked in random order or each crystal domain grows random layers. No symmetry would exist in this case and the crystal would not be defined by a lattice constant (*d*-spacing), that is, no clear diffraction pattern.

Besides the stacking order, the offset orientation also matters. Figure 6h, i illustrates the subtle structural difference when a change in the offset orientation occurs. The *p*6 monolayer can work as a mirror plane in between two continuous layers, as captured in a cluster from our MD simulation as evidence for such structural complexity (Fig. 6j). This fact reminds us that despite the cubic Type II can be interpreted as a layered structure, only when the offset orientation is consistent across layers, the formed phase possesses the conventional cubic $Fd\overline{3}m$ symmetry. The possibility of changing offset orientation highlights the layered inter-growth structure complexity into another level, and it also indicates the twinned Type II crystals (*p*6 monolayer as mirror surface), which expands the possible morphologies one could observe macroscopically^31^.

Focusing on the 5^12^ cages and hexagonal rings, one can notice a common pattern in the interlayer of Types II and HS-I (gray lines in Fig. 6e, f), which indicates another pathway linking these two structures. A simple geometrical calculation also gives further evidence to this possibility: 9.988 Å/$\sin 54.{7}^{\circ }$ = 12.2 Å, which is consistent with the *d*-spacing in Type HS-I, 12.1 Å. Figure 6k illustrates how the Types II and HS-I fuse together through the same arrangement of the interlayer structure, and Fig. 6l,m shows an example captured from our MD simulations. As described in the previous section, a complex H-bond network can be generated by considering the building blocks, this special pathway can be derived from the combination of pyramid building blocks and PC3 building blocks (see detail structure in Supplementary Movie 2). In addition, as discussed in the former section, Type HS-I can transit to Type I. Viewing from the Fig. 6m, its right surface can smoothly transit to AUC of Type I (Supplementary Fig. 11). Type HS-I is a bridge whereby the Types I and II can be linked, which is the rationale for the 5^12^6^3^ cages found in the interface between Types I and II clathrate hydrates in previous studies^32,33^, however, there is no evidence in the literature that anyone has realized this pathway is made possible via the Type HS-I.

Except for Type H, all the other crystals discussed in this work have counterparts as FK phases. Considering close-packed mode, the topological dual of Type H network corresponds to the CaCu_5_ prototype in the intermetallic structure, which is considered quasi-FK phases^22^. Natarajan and Van der Ven^34^ demonstrated the hexagonal close-packed (HCP) can easily transform to the CaCu_5_ prototype via one layer’s atoms slight collective displacement. From the viewpoint of topological duals, the pathways from Type H to other clathrate hydrates networks described in this work provide further diverse pathways from HCP to TCP, which is useful for a deeper understanding the structural transition in continuous solid phase of other materials.

## Discussion

We report an alternative way to understand clathrate hydrates, that is, diverse crystalline H-bonded networks can be generated through assembling of one common building block. Based on the building blocks proposed in this work, we unveil hidden symmetries contained in clathrate hydrates crystals and intrinsic relations among them. The alternative interpretations of structures not only clarify the richness of pathways linking different crystals, but further derive some unexpected structure complexities, supported by evidence from MD simulations. Results also suggest a continuous nucleation and crystal growth mechanism, that is, one ordered structure could be a seed for another structure to consecutively grow without forming a structurally incompatible interface. Except for the Type H, the H-bonded network of clathrate hydrates discussed in this work are the topological duals of FK phases, including three of their cornerstone structures (*A*15, *C*15, *Z*). In the light of this dual relation, the assembly of building blocks described in this work automatically provides a description of the space-filling of TCP networks. Thus, our results from clathrate hydrates can be directly extrapolated to other TCP ordered materials and detail a rational phase transition mechanisms in the solid state. These results demonstrate the structural richness of clathrate hydrates, which being connected to the Frank–Kasper phases, offer opportunity to apply these same ideas to other order materials. For clathrate hydrates in particular, this opens venues to explore structures that may have never been considered before, providing further diverse options for energy/gas storage. Finally, we argue that the significance of clathrate hydrates research is more than just in the energy areas. Based on the simple and common patterns observed, this class of natural substances contains clues for a deeper understanding of other apparently unrelated materials across different physical scales.

## Methods

### MD simulation method

Our MD simulations were performed using open-source GROMACS 2020^35,36^. All the simulations started from completely disordered two phases separated configurations with the guest molecules (methane and propane molecules described by OPLS-UA model^37^) in a nano-bubble surrounded by water molecules (described by TIP4P/ice model^38^). The system contained 3672 water molecules, 432 methane molecules, and 54 propane molecules. The simulation box considered was a cubic box 5 nm in length. The initial configuration were obtained by melting 3 × 3 × 3 Type II crystal. We performed 20 repeat NPT simulations at 250 K and 1000 bar to capture the nucleation, each 3000 ns long. Briefly, this set of conditions was obtained by performing successive simulations, if nucleation was not observed at a given set of conditions, then the pressure was increased in the following set of simulations; more details of this procedure and simulation parameters can be found in our previous paper^39^. All of the captured ordered clusters in this work formed spontaneously without any constraints. In total, all simulations conducted during this study required about 4000 h (using 72 CPUs in parallel per simulation) on the High Performance Computing cluster at the Colorado School of Mines.

### Analysis

Hydrogen bonds and water cages were analyzed via custom-developed codes in Python. The ordered cluster are captured semi-automatically by looking at trajectory frame by frame and pick up cages manually. The fast Fourier transform is conducted by GIXStapose, an open source Python package, it can be download at, https://github.com/cmelab/GIXStapose. The 2D-FFT image was obtained by projecting the ordered cluster along high-symmetry axes and then applying a fast Fourier transform.

## Supplementary information

Supporting Information

Description of Additional Supplementary Files

Supplementary Movie 1

Supplementary Movie 2

## Data Availability

The data that support the findings of this study are available from the corresponding author upon request.
