# Supplementary material for: Topological dual and extended relations between networks of clathrate hydrates and Frank-Kasper phases
Source: Nat Commun. 2023 Feb 3;14:596. doi: 10.1038/s41467-023-36242-4 (PMC9898525; doi:10.1038/s41467-023-36242-4)
Supplement: Supplementary file 1 — Supporting Information [file 41467_2023_36242_MOESM1_ESM.pdf]

Supplementary Information for Topological dual and extended relations between networks of clathrate hydrates and Frank-Kasper phases

Yong Chen,[1,2,3,4,5] Satoshi Takeya,[6] and Amadeu K. Sum[*,1]

[1]*Phases to Flow Laboratory, Chemical & Biological Engineering Department, Colorado School of Mines, 1500 Illinois Street, Golden, 80401, Colorado, United States*

[2]*Guangzhou Institute of Energy Conversion, Chinese Academy of Sciences, No.2, Nengyuan Road, Wushan, Tianhe District, Guangzhou, 510640, Guangdong, P. R. China*

[3]*CAS Key Laboratory of Gas Hydrate, Guangzhou, 510640, Guangdong, P. R. China*

[4]*Guangdong Provincial Key Laboratory of New and Renewable Energy Research and Development, Guangzhou, 510640, Guangdong, P. R. China*

[5]*State Key Laboratory of Natural Gas Hydrate, Beijing, 100028, P. R. China*

[6]*National Metrology Institute of Japan (NMIJ), National Institute of Advanced Industrial Science and Technology (AIST), Central 5, Higashi 1-1-1, Tsukuba, 305-8565, Ibaraki, Japan*

* Corresponding author(s). E-mail(s): asum@mines.edu

Supplementary Note 1. Discussion on topological dual and steric connectivity

Taking $A15$ of Frank-Kasper (FK) phases and Type I clathrate hydrates for example, as shown in Supplementary Fig. 1a, applying Voronoi tessellation to tetrahedral close-packed (TCP) nodes will generate a different form of network. This network is composed by hydrogen-bonded (H-bonded) water molecules forming clathrate hydrates (Supplementary Fig. 1a); a 5^{12} cage on the corner of the Type I clathrate hydrate unit cell shows the detailed arrangement of the water molecules. TCP nodes and H-bond network are two sides of the same coin. Considering the connectivity of H-bonds is equivalent to considering the arrangement of the TCP nodes.

The network of TCP nodes are generally described by Coordination Number (CN) polyhedrons and their geometry is shown in Fig. 1. The surrounding nodes of one CN polyhedron are the center or surrounding nodes for another CN polyhedron, that means two CN polyhedrons can be connected by sharing a node, an edge, a triangular face, or a region. As show in Supplementary Fig. 1b, the intersection of CN12 and CN14 in $A15$ phase is a region (bounded by magenta nodes and two red central nodes). The complex connectivity among CN polyhedrons makes it intractable in understanding detailed 3D structures. While the connectivity among their topological duals, Voronoi cells, is much simpler and much more defined. Mindful that these Voronoi cells actually are H-bond cages, their connectivity must satisfy the connectivity of hydrogen-bonded water molecules. The maximum number of hydrogen bonds that a water molecule can form is four, donating two via its two hydrogen atoms and accepting two via its oxygen atom. Thus, when two H-bond cages are connected, they must be connected by sharing a full face, that is the only way. If two H-bond polyhedrons connect by sharing one node (a water molecule) or one edge (a H-bond), it means the total edges converging at the shared nodes exceeds four, which is impossible for H-bonded water molecules. One can use a 2D image as an analogy, as show in Supplementary Fig. 1c,d.

To obtain a points array like Supplementary Fig. 1c, one can try to directly consider the arrangement pattern of these points. On the other hand, one can consider a framework composed of a series of triangles, as shown in Supplementary Fig. 1d. In this case, one only needs to place a node at the center of each triangle, then the same points array in Supplementary Fig. 1c automatically appears. Considering TCP nodes directly generate the points array in Supplementary Fig. 1c, and considering H-bond cages with the method shown in Supplementary Fig. 1d.

Owing to the complex connectivity among CN polyhedrons, some methods were developed for a better interpretation and exploration of the TCP network. The major network/skeleton associated with layers of nets is the best known method. As shown in Fig. 1 in the manuscript, there are two kinds of nodes in the CN polyhedrons, 5-fold and 6-fold. The lines connecting the 6-fold nodes are called the major network/skeleton of FK phases. Supplementary Fig. 2a shows the major network (green lines) of $A15$ phase. In this method, the TCP nodes are sliced into layers of nets formed by the major network. Supplementary Fig. 2b shows the nets that establishes $A15$ phase, however, it is not straightforward to consider and understand the correlations across layers, while the basic building block (BBB) provide this information, as shown in Supplementary Fig. 2a, where the PC1 building block shows the connectivity among different layers of net. We see major lines penetrate the hexagonal rings of the BBB, so it also provides a way to geometrically combine major network and CN12 nodes. Moreover, for the major network/skeleton method, different FK phases have their own set of nets, which also makes it difficult to understand the structural relations among diverse FK phases. The BBB perspective has advantage in this aspect, since structures are considered through one common building block, as discussed in the manuscript. One can find more information about major network of other type FK phases,[1,2] and other interpretation methods[3–6] of FK phases in other studies.

Overall, a simpler and much more defined connectivity among H-bond cages makes it helpful for a better understanding of the detailed 3D structures. Based on this point, the

building blocks proposed in this work (through deconstruction of the H-bond network of clathrate hydrates) provide a simplified and unified way to understand intricate 3D networks of FK phases and clathrate hydrates, just like auxiliary lines used in solving complex geometric problems, which reveal the intrinsic relations and pathways linking diverse crystals.

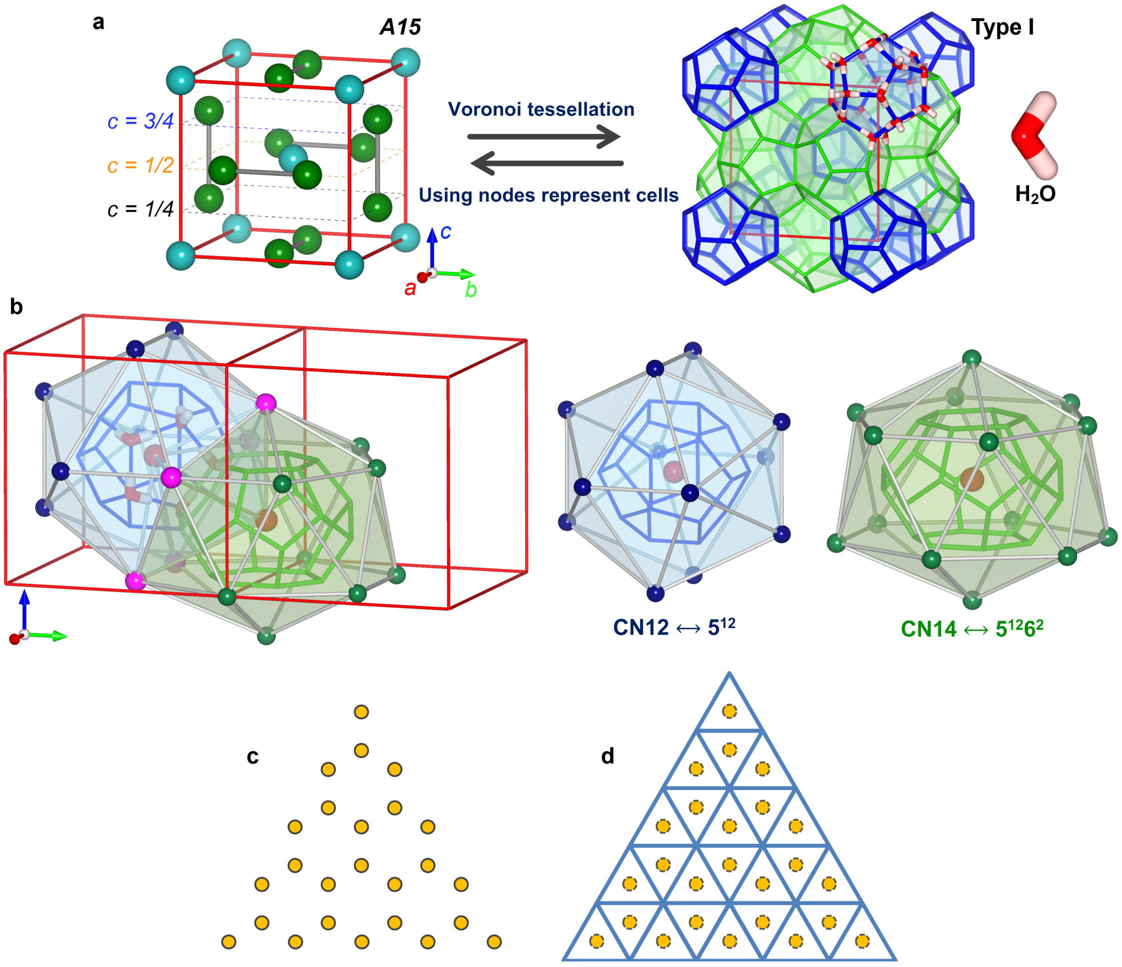

Supplementary Fig. 1 Topological duality and connectivity. **a** Illustration of topological dual relationship between TCP and H-bond network of clathrate hydrates, using *A*15 of FK phases and Type I clathrate hydrates as examples. The cyan and dark green spheres represent central nodes of CN12 and CN14, respectively. **b** Steric connectivities of the CN12 and CN14 in *A*15 phase, and their topological duals, 5^{12} and $5^{12}6^2$ cages. The central node is displayed in red, and the surrounding nodes of CN12 and CN14 are dark blue and dark green, respectively. The magenta nodes are shared by CN12 and CN14. **c,d** A 2D image as an analogy to illustrate the strategy of considering the TCP nodes and H-bond cages.

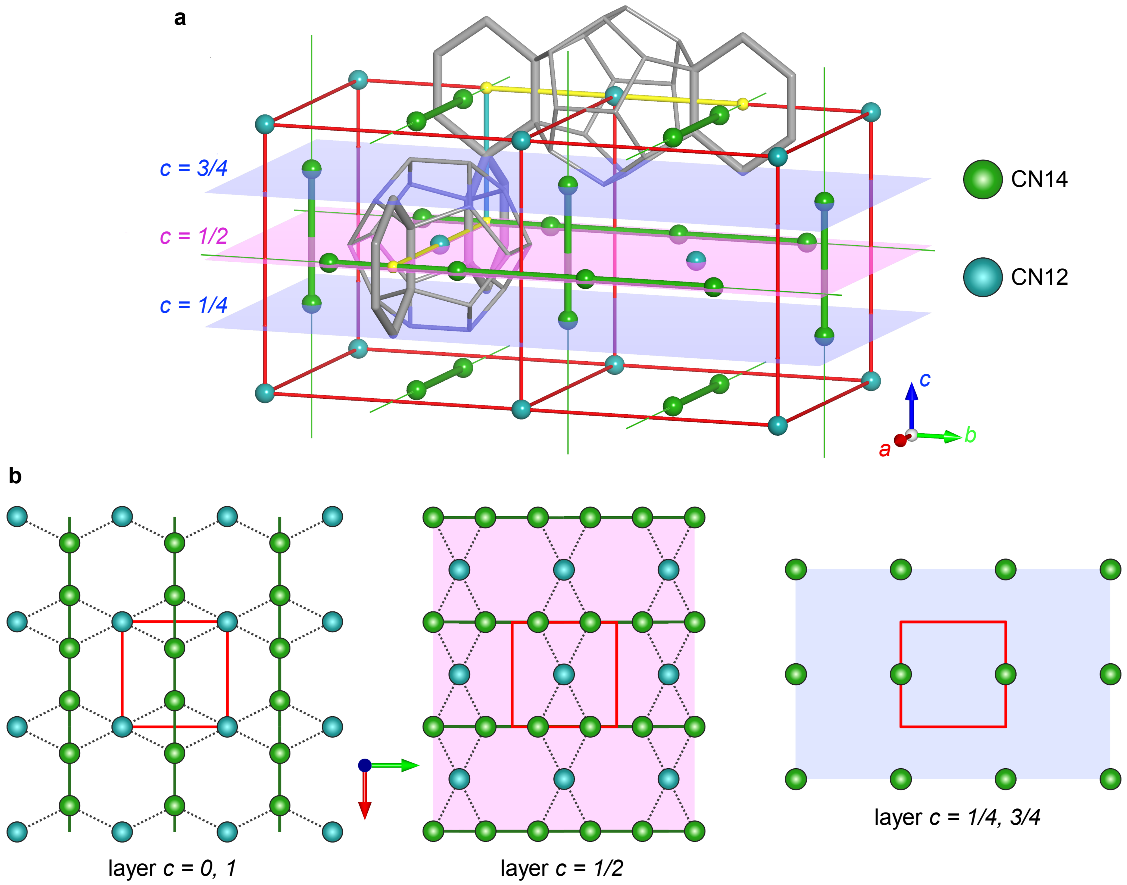

Supplementary Fig. 2 Major network and basic building block. a *A*15 of FK phases, the cyan and dark green spheres represent central nodes of CN12 and CN14, respectively. Green lines indicate the major networks/skeleton. The BBBs are displayed in grey for clarity. **b** Superposition of a set of nets constitute *A*15 phase. Red square indicates the unit cell box, green lines indicate the major network.

Supplementary Note 2. Distortion of Basic building block

The BBB is not exactly the same in different clathrate hydrates in terms of geometric parameters. In Supplementary Fig. 3a, we align the BBB in Type I (red) and Type HS-I (blue) to illustrate the slightly differences and distortions. The 5^{12} cages in clathrate hydrates are not perfect pentagonal dodecahedra, with some pentagonal rings not on the same flat plane, as shown in Supplementary Supplementary Table 2 listing their symmetry in different structures. While hydrogen bonds are flexible in terms of distance and angle among water molecules, certain distortion and adjustment are allowed when building blocks assemble to form complex structures – a cluster shown in Fig. 5 in the manuscript is a good example. It is noteworthy that CN polyhedrons are not exactly the same among FK phases as well, as the structural stability allows a certain freedom to the atoms' positions, also a key feature of FK phases.[1,2,7]

Supplementary Table 1 Crystal parameters of clathrate hydrates. The cages in the "Cell content" column have consistent color code used in the Supplementary Fig. 5h (see below).

Clathrate hydrates	FK counterpart	Space Group	Cell dimensions (Å)	Cell content
Type I	*A*15	$Pm\bar{3}n$ Cubic	$a = b = c = 12.03$ Ref. 8	$[2{\cdot}(5^{12}){+}6{\cdot}(5^{12}6^{2})] \cdot 46\ H_2O$
Type II	*C*15	$Fd\bar{3}m$ Cubic	$a = b = c = 17.3$ Ref. 9	$[16{\cdot}(5^{12}){+}8{\cdot}(5^{12}6^{4})] \cdot 136\ H_2O$
Type H	–	$P6/mmm$ Hexagonal	$a = b = 12.2, c = 10.1$ Ref. 10	$[3{\cdot}(5^{12}){+}1{\cdot}(5^{12}6^{8}){+}2{\cdot}(4^{3}5^{6}6^{3})] \cdot 34\ H_2O$
Type HS-I	*Z*	$P6/mmm$ Hexagonal	$a = b = 11.85, c = 12.14$ Ref. 11	$[3{\cdot}(5^{12}){+}2{\cdot}(5^{12}6^{2}){+}2{\cdot}(5^{12}6^{3})] \cdot 40\ H_2O$
Type TS-I	*Sigma*	$P4_2/mnm$ Tetragonal	$a = b = 23.04, c = 12.07$ Ref. 12	$[10{\cdot}(5^{12}){+}16{\cdot}(5^{12}6^{2}){+}4{\cdot}(5^{12}6^{3})] \cdot 172\ H_2O$

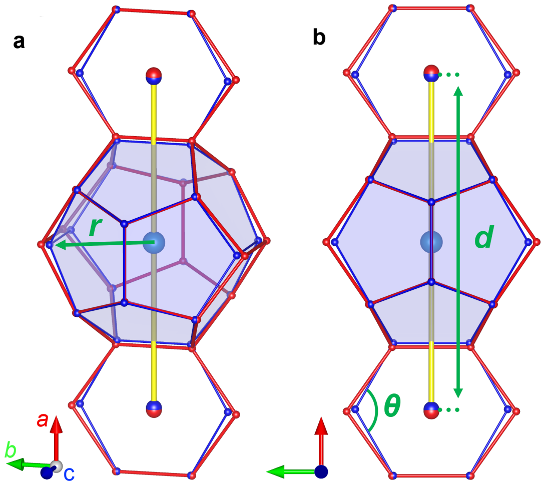

Supplementary Fig. 3 Basic building block in different clathrate hydrates structures. a Geometric difference of basic building block in Type I (red) and Type HS-I (blue). **b**, View **(a)** along c direction. r is the distance to the center of 5^{12}; d is the length of centerline; θ is angle in hexagonal ring. The detailed parameters in different crystals are listed in the Supplementary Table 2.

Supplementary Table 2 Geometry parameters of basic building block in different clathrate hydrates structures. r is the distance to the center of 5^{12}; d is the length of centerline; θ is angle in hexagonal ring, see Supplementary Fig. 3. These data were collected from crystallographic data; corresponding reference can be found in Supplementary Table 1.

Basic building block in	Symmetry of 5^{12}	d (Å)	Range of r (Å)	Range of θ (°)
Type I	$m\overline{3}$	12.03	3.82 – 3.97	109 –125
Type II	$m\overline{3}$	12.23	3.74 – 3.95	119.89
Type H	mmm	12.2	3.78 – 3.94	120
Type HS-I	mmm	11.85	3.68 – 4.03	120
Type TS-I	mm<br>m	12.26	3.71 – 4.02<br>3.77 – 3.96	117 – 123

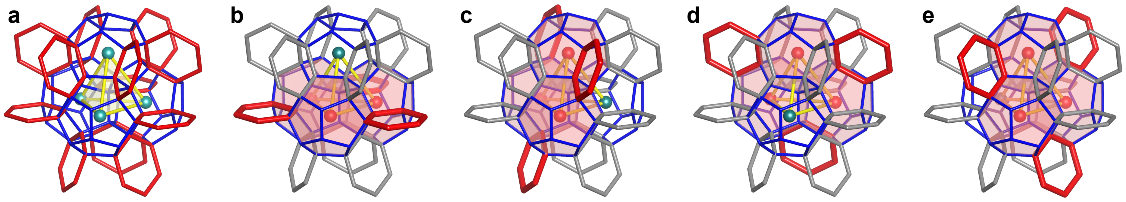

Supplementary Fig. 4 Relationship between the pyramid building block and the PC2 building blocks. **a** Pyramid building block. **b–e** Interlacing of PC2 forming the pyramid building block. Any three 5^{12} cages satisfy the PC2, as highlighted in red.

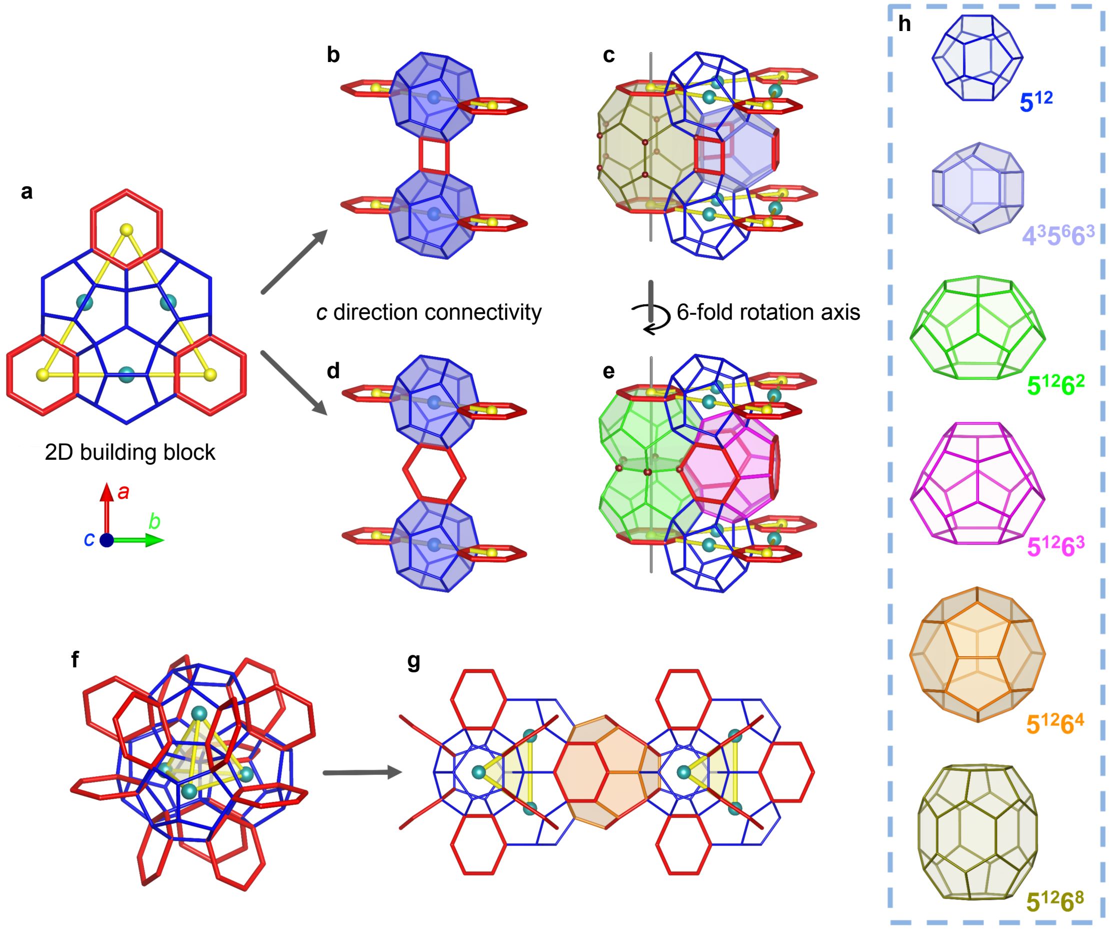

Supplementary Fig. 5 Building blocks and other elementary cages. **a** View PC2 along *c* direction. **b,c** The PC2 vertically connected through H-bond forming a square face, the six-fold rotation symmetry results in the $5^{12}6^{8}$ and $4^{3}5^{6}6^{3}$ cages. **d,e** The PC2 vertically connected through a hexagonal ring, and the six-fold rotation symmetry generates the $5^{12}6^{2}$ and $5^{12}6^{3}$ cages. It should be noted that the square face means 5^{12} cages are directly H-bonded, formed without introducing other nodes (water molecules). **f,g** The translational duplication of the pyramid building blocks forms the $5^{12}6^{4}$ cage. **h** The geometry of elementary cages in clathrate hydrates. The color code of cages in **(a–g)** are consistent with **(h)**.

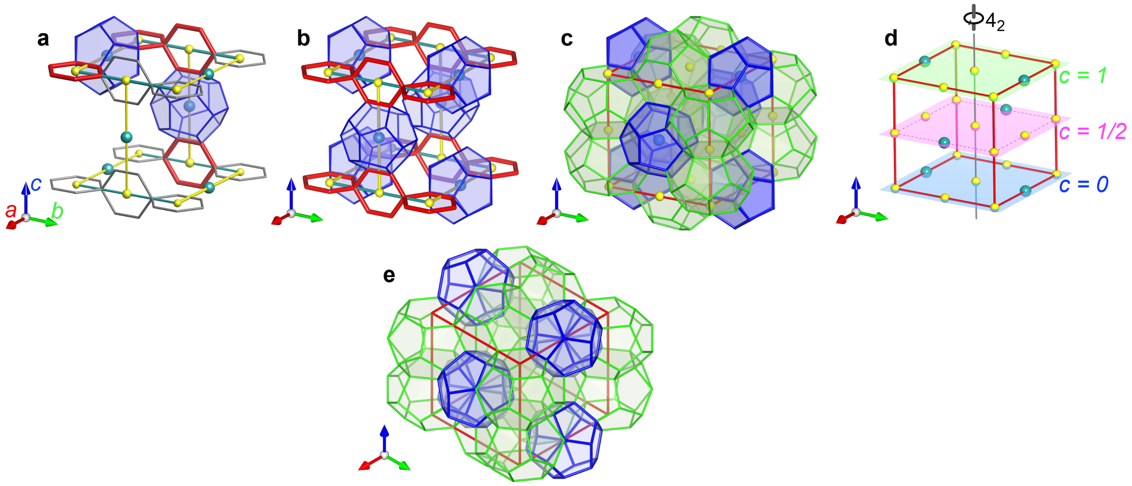

Supplementary Fig. 6 The cube building block and 4_2 screw symmetry. a,b PC1 connectivity establishes the cube building block, some structures are hidden and displayed in grey for clarity. **c** The complete version of alternative unit cell of Type I. **d** Only shows the lattice nodes to manifest the 4_2 screw symmetry. The yellow and cyan spheres represent centers of hexagonal rings and 5^{12} cages. **e** View along the body diagonal direction, which shows no 3-fold symmetry along this direction.

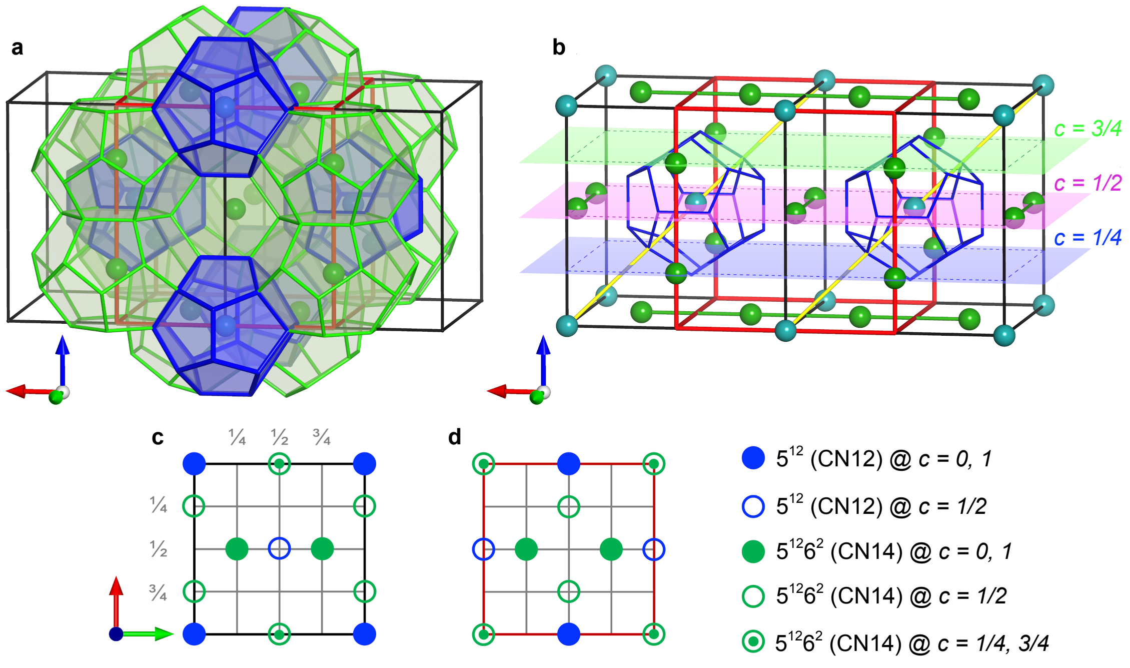

Supplementary Fig. 7 Relationship between conventional unit cell and alternative unit cell of Type I clathrate hydrates. **a** The alternative unit cell (AUC) is the conventional unit cell (CUC) shifted half unit cell. The black and red box is CUC and AUC of Type I, respectively. Blue cages are 5^{12} and green cages are $5^{12}6^2$. **b** From the perspective of tetrahedrally close-packed FK structure ($A15$ phase). Cyan and green spheres represent central nodes of CN12 and CN14, respectively. The yellow lines in indicates the $\bar{3}$ axes of $Pm\bar{3}n$ unit cell (along body diagonal). **c** 2D version of conventional unit cell, $Pm\bar{3}n$. **d** 2D version of alternative unit cell, $P4_2/mmc$.

Supplementary Table 3 Parameters of conventional unit cell and alternative unit cell of Type I structure. The cages in the "Cell content" row have consistent color code used in the Supplementary Fig. 5h.

Type I / $A15$	Conventional unit cell	Alternative unit cell
Space group	$Pm\bar{3}n$<br>*Cubic*	$P4_2/mmc$<br>*Tetragonal*
Cell dimensions (Å)	$a = b = c = 12.03$<br>$\alpha = \beta = 90°$	$a = b = c = 12.03$<br>$\alpha = \beta = \gamma = 90°$
Number of water	46	46
Cell content (cages)	$2{\cdot}(5^{12}){+}6{\cdot}(5^{12}6^2)$	$2{\cdot}(5^{12}){+}6{\cdot}(5^{12}6^2)$

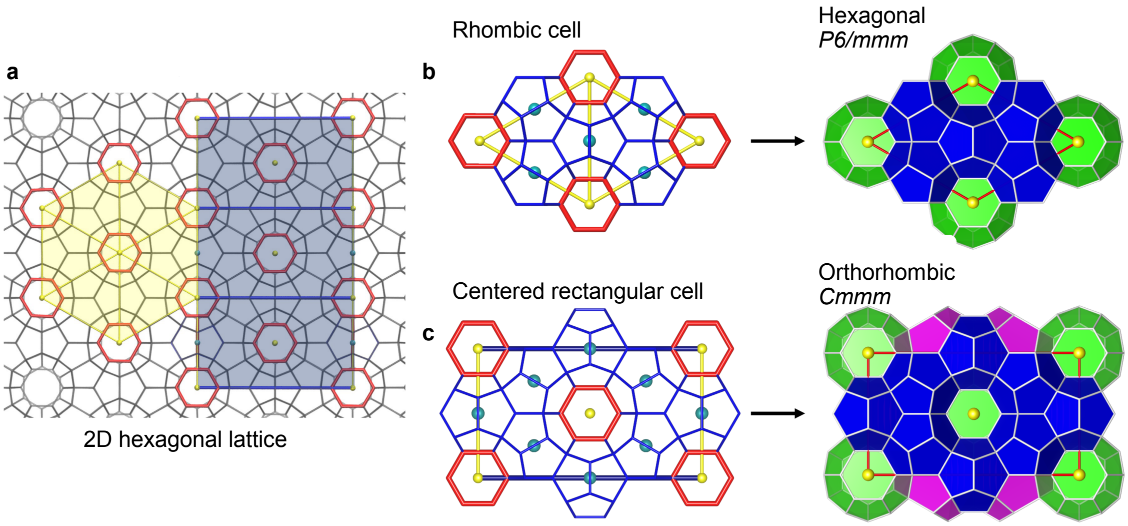

Supplementary Fig. 8 The alternative unit cell of Type HS-I. a The primitive 2D hexagonal lattice can be represented by the rhombic cell or the centered rectangular cell. The yellow region indicates the six-fold symmetry in the hexagonal lattice, which is represented by rhombic cell **(b)**. And blue region indicates the centered rectangular version, as shown in **(c)**. **b** The rhombic cell leads to the $P6/mmm$ unit cell. **c** The centered rectangular cell leads to the $Cmmm$ unit cell.

Supplementary Table 4 Parameters of conventional unit cell and alternative unit cell of Type HS-I structure. The cages in the "Cell content" row have consistent color code used in the Supplementary Fig. 5h.

Type HS-I / Z	Conventional unit cell	Alternative unit cell
Space group	$P6/mmm$<br>$Hexagonal$	$Cmmm$<br>$Orthorhombic$
Cell dimensions (Å)	$a = b = 11.85, c = 12.136$<br>$\alpha = \beta = 90°, \gamma = 120°$	$a = 11.85, b = 20.525, c = 12.136$<br>$\alpha = \beta = \gamma = 90°$
Number of water	40	80
Cell content (cages)	$3{\cdot}(5^{12})+2{\cdot}(5^{12}6^2)+2{\cdot}(5^{12}6^3)$	$6{\cdot}(5^{12})+4{\cdot}(5^{12}6^2)+4{\cdot}(5^{12}6^3)$

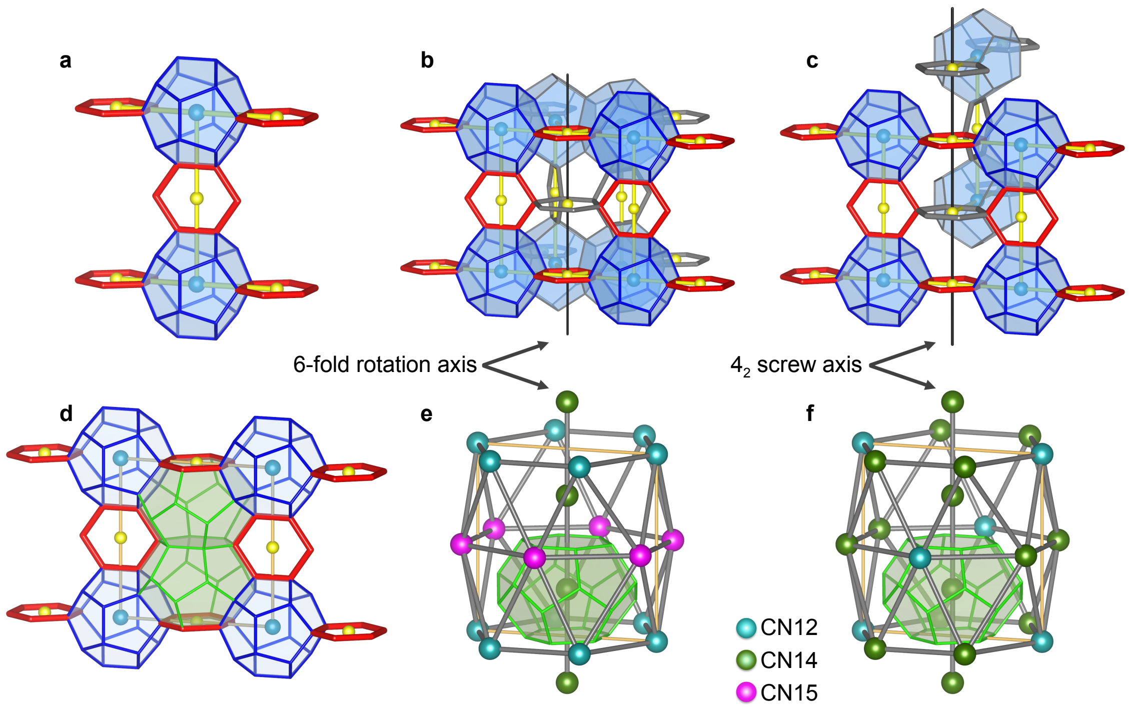

Supplementary Fig. 9 Intrinsic relations between Type I and HS-I networks. a-c The same building block generates Type HS-I and Type I with different rotation operation, the six-fold rotation axis builds the former one and the 4_2 screw axis generates the latter one. Both of these two rotation symmetry results in a column of $5^{12}6^2$ cages forming a common cross-section, as show in **(d)**. **e,f** Corresponding figure of **(b,c)** from the viewpoint of tetrahedrally close-packed structures. **(e)** and **(f)** are the hexagonal anti-prisms configuration present in Z and $A15$ phases, respectively. The cyan, green, and magenta spheres represent central nodes of CN12, CN14, and CN15. The yellow lines indicate the same plane. In clathrate hydrates system, this plane corresponds to the structure shown in **(d)**.

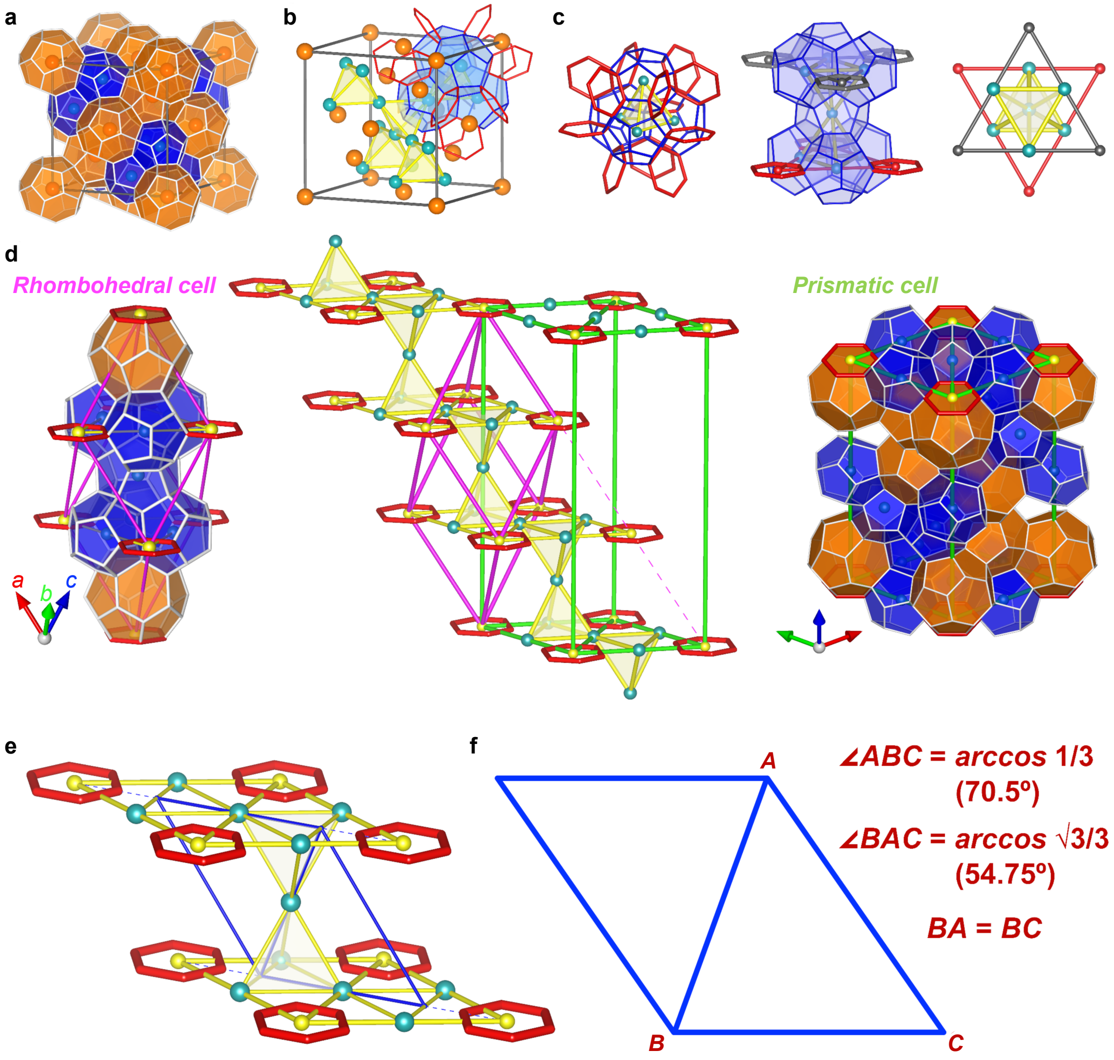

Supplementary Fig. 10 Alternative unit cell of Type II. a,b Conventional cubic unit cell of Type II structure ($Fd\bar{3}m$). 5^{12} and $5^{12}6^4$ are displayed in blue and orange, respectively. Cages are hidden in **(b)** for highlighting the pyramid building blocks. **c** Inverted connected pyramid building blocks. Hexagonal rings are displayed in different color (red and grey) to show the relationship between two layers, that is, the red layer six-fold axes coincide with grey layer three-fold axes, and vice-versa. **d** A series of $p6$ layers (represented by R-A building block) generated by the inverted connected pyramid building blocks. Two versions of the unit cell can be obtained from the special offset stacking of $p6$ layers. Magenta and green lines indicate the rhombohedral and prismatic cell boundary, respectively. **e,f** Geometric parameters in the offset layers. Based on the pyramid building block, one can easily infer the offset angle in the layered Type II is 54.7° (angle between an edge and a face in the regular tetrahedron)

Supplementary Table 5 Parameters of conventional unit cell and alternative unit cell of Type II structure. The cages in the "Cell content" row have consistent color code used in the Supplementary Fig. 5h.

Type II / $C15$	Conventional unit cell	Alternative unit cell rhombohedral cell version	Alternative unit cell prismatic cell version
Space group	$Fd\bar{3}m$ *Cubic*	$Rd\bar{3}m$ *Trigonal*	$Rd\bar{3}m$ *Trigonal*
Cell dimensions (Å)	$a = b = c = 17.3$ $\alpha = \beta = \gamma = 90°$	$a = b = c = 12.23$ $\alpha = \beta = \gamma = 60°$	$a = b = 12.23, c = 29.96$ $\alpha = \beta = 90°, \gamma = 120°$
Number of water	136	34	102
Cell content (cages)	$16{\cdot}(5^{12})+8{\cdot}(5^{12}6^4)$	$4{\cdot}(5^{12})+2{\cdot}(5^{12}6^4)$	$12{\cdot}(5^{12})+6{\cdot}(5^{12}6^4)$

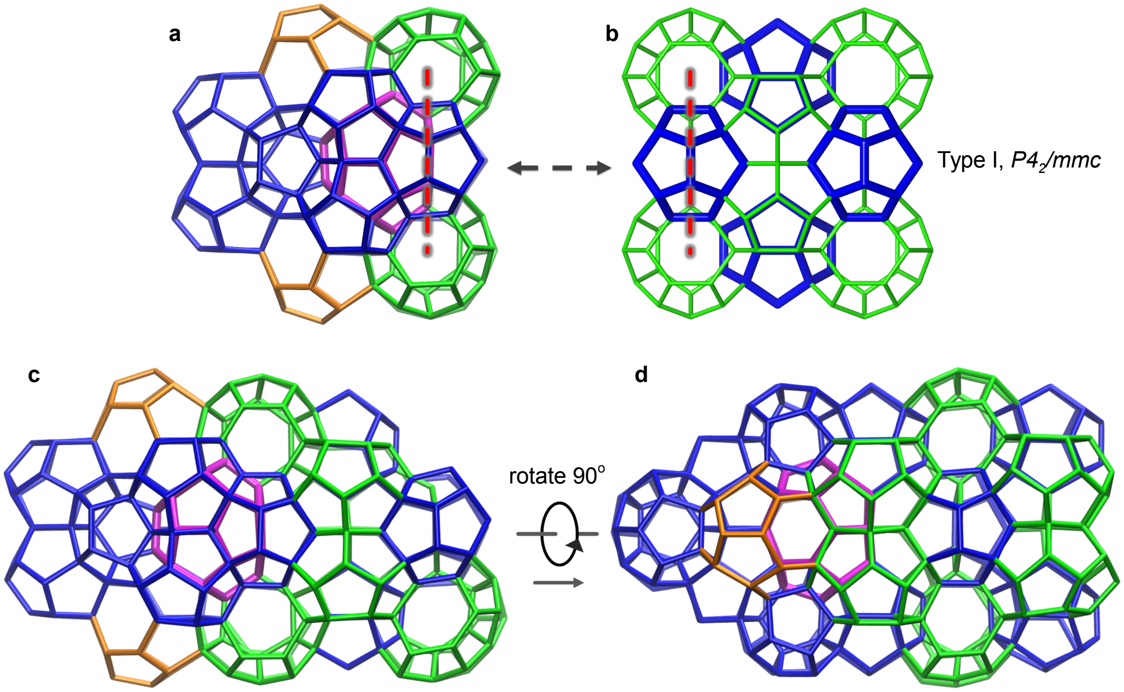

Supplementary Fig. 11 Types I and II are linked via Type HS-I. a,b The right surface of the cluster shown in Fig. 6h can transit to $P4_2/mmc$ unit cell of Type I, which follows the mechanism described in Types I and HS-I pathway section. The red dash lines highlight the same structure. **c,d** An example captured in MD simulation, although the $P4_2/mmc$ unit cell is incomplete.

Supplementary references

(1) Shoemaker, D., Shoemaker, C. & Jaric, M. Introduction to quasicrystals (1988).

(2) Frank, F. C. & Kasper, J. S. Complex alloy structures regarded as sphere packings. I. Definitions and basic principles. *Acta Cryst. Sect. A* **11**, 184–190 (1958) .

(3) Andersson, S. Structures related to the β-Tungsten or Cr_3Si structure type. *J. Solid State Chem.* **23**, 191–204 (1978) .

(4) Magruder, B. R. & Dorfman, K. D. The C36 Laves phase in diblock polymer melts. *Soft Matter* **17**, 8950–8959 (2021) .

(5) Nelson, D. R. Order, frustration, and defects in liquids and glasses. *Phys. Rev. B* **28**, 5515 (1983) .

(6) Travesset, A. Nanoparticle superlattices as quasi-Frank-Kasper phases. *Phys. Rev. Lett.* **119**, 115701 (2017) .

(7) Ungar, G. & Zeng, X. Frank-Kasper, quasicrystalline and related phases in liquid crystals. *Soft Matter* **1**, 95–106 (2005) .

(8) Mcmullan, R. K. & Jeffrey, G. A. Polyhedral Clathrate Hydrates. IX. Structure of Ethylene Oxide Hydrate. *J. Chem. Phys.* **42**, 2725–2732 (1965) .

(9) Mak, T. C. W. & McMullan, R. K. Polyhedral Clathrate Hydrates. X. Structure of the Double Hydrate of Tetrahydrofuran and Hydrogen Sulfide. *J. Chem. Phys.* **42**, 2732–2737 (1965) .

(10) Ripmeester, J. A., Tse, J. S., Ratcliffe, C. I. & Powell, B. M. A new clathrate hydrate structure. *Cah. Rev. The.* **325**, 135–136 (1987) .

(11) Kosyakov, V. I., Shestakov, V. A. & Solodovnikov, S. F. Calculation of the gas hydrate HS-1 framework structure and its energy estimation. *J. Struct. Chem.* **34**, 810–813 (1994) .

(12) Udachin, K. A., Enright, G. D., Ratcliffe, C. I. & Ripmeester, J. A. Structure, Stoichiometry, and Morphology of Bromine Hydrate †. *J. Am. Chem. Soc.* **119**, 11481–11486 (1997) .
