# Supplementary material for: Topological dual and extended relations between networks of clathrate hydrates and Frank-Kasper phases
Source: Nat Commun. 2023 Feb 3;14:596. doi: 10.1038/s41467-023-36242-4 (PMC9898525; doi:10.1038/s41467-023-36242-4)
Supplement: Supplementary file 2 — Description of Additional Supplementary Files [file 41467_2023_36242_MOESM2_ESM.pdf]

File name: Supplementary Movie 1
Description: Visualization of nucleation process of the cluster shown in Fig. 5a in the manuscript. a The ordering process of water molecules. **b** Only H-bonds are shown and clathrate hydrates cages are displayed by different colors. There are a total of 121 cages in this cluster, 1244 water molecules in total. The number of 5^{12} (blue), $5^{12}6^{2}$ (green), and $5^{12}6^{3}$ (magenta) are 43, 67, and 11, respectively.

File name: Supplementary Movie 2
Description: Illustration of the special pathway between Types II and HS-I. a The pyramid building block combines with PC3, which clarifies this special pathway. **b** The detail structure after showing other elementary cages.
